# Milk Intake and Total Dairy Consumption: Associations with Early Menarche in NHANES 1999-2004

**DOI:** 10.1371/journal.pone.0014685

**Published:** 2011-02-14

**Authors:** Andrea S. Wiley

**Affiliations:** Department of Anthropology, Program in Human Biology, Indiana University, Bloomington, Indiana, United States of America; University of Delaware, United States of America

## Abstract

**Background:**

Several components of dairy products have been linked to earlier menarche.

**Methods/Findings:**

This study assessed whether positive associations exist between childhood milk consumption and age at menarche or the likelihood of early menarche (<12 yrs) in a U.S sample. Data derive from the National Health and Nutrition Examination Survey (NHANES) 1999–2004. Two samples were utilized: 2657 women age 20–49 yrs and 1008 girls age 9–12 yrs. In regression analysis, a weak negative relationship was found between frequency of milk consumption at 5–12 yrs and age at menarche (daily milk intake β = −0.32, *P*<0.10; “sometimes/variable milk intake” β = −0.38, *P*<0.06, each compared to intake rarely/never). Cox regression yielded no greater risk of early menarche among those who drank milk “sometimes/varied” or daily vs. never/rarely (HR: 1.20, *P*<0.42, HR: 1.25, *P*<0.23, respectively). Among the 9–12 yr olds, Cox regression indicated that neither total dairy kcal, calcium and protein, nor daily milk intake in the past 30 days contributed to early menarche. Girls in the middle tertile of milk intake had a marginally lower risk of early menarche than those in the highest tertile (HR: 0.6, *P*<0.06). Those in the lowest tertiles of dairy fat intake had a greater risk of early menarche than those in the highest (HR: 1.5, *P*<0.05, HR: 1.6, *P*<0.07, lowest and middle tertile, respectively), while those with the lowest calcium intake had a lower risk of early menarche (HR: 0.6, *P*<0.05) than those in the highest tertile. These relationships remained after adjusting for overweight or overweight and height percentile; both increased the risk of earlier menarche. Blacks were more likely than Whites to reach menarche early (HR: 1.7, *P*<0.03), but not after controlling for overweight.

**Conclusions:**

There is some evidence that greater milk intake is associated with an increased risk of early menarche, or a lower age at menarche.

## Introduction

The age at which a female reaches sexual maturity represents a critical transition in her life history. While to a large degree the timing of menarche is under genetic control [1], [2], [3], environmental signals of resource availability or stress, among others, may accelerate or delay this life history shift from somatic to reproductive investment. Under conditions of food scarcity, it may make sense to delay menarche until sufficient energy resources are available to support reproduction [4], [5], [6], while familial or local social stressors appear to accelerate menarche when social conditions are unstable and future survival is uncertain [7], [8], [9], [10]. A downward secular trend in age at menarche has occurred when food resources have become abundant and life-threatening childhood infections have diminished, such that over the course of the twentieth century age at menarche declined from over age 16 yrs to below 13 yrs in industrialized countries, and similar trends are evident in contemporary developing countries [1], [11], [12], [13].

Nutrient intake and storage during childhood may influence the timing of menarche through hormones such as leptin and insulin, and growth factors such as Insulin-like Growth Factor I (IGF-I), all of which are involved in the regulation of growth and maturation [1], [14], [15]. Chronic energy undernutrition has been linked to later age at menarche [4], [11], [16], while overnutrition, characterized by high BMI, has been linked to earlier onset of menarche [17], [18], [19], [20], [21], [22]. The effect of childhood nutrition on menarche is contingent on the physiological template established in utero and the infant period, with lower birth weight and rapid growth in early childhood associated with earlier menarche [15], [20], [22], [23], [24], [25]. There is variation in age at menarche among population groups in the U.S., with African Americans averaging a younger age at menarche than Whites [17], [26], [27], [28], while Mexican-American girls have average ages at menarche between those of Blacks and Whites [28]. This variation is not well-understood, but is likely to stem from differences in experiences with the factors just outlined [29]. There may also be population genetic differences that contribute to variation in age at menarche.

While overall nutritional status mediates age at menarche, there has been additional research relating specific dietary components to this outcome. Low fiber and monounsaturated fatty acid intake [30], high calcium [31], animal protein [32], [33], [34], and animal fat intake [30], [35] each have been associated with a lower age at menarche, although not consistently across studies [36], [37]. The latter three nutrients are common to milk and other dairy products, suggesting that the consumption of these particular foods may influence the timing of sexual maturation. Milk supplementation studies designed to assess effects on bone mineralization among peri-menarcheal girls have generally not found effects on age at menarche [38], [39]. Chinese girls receiving milk supplements over 2 years were not more likely to reach menarche during the supplementation period than girls in the control group (48.3% compared with 40.1%; *P* = 0.09), and there were no significant differences in age at menarche between supplementation and control groups 3 years after the supplementation ended, when over 97% of study participants had reached menarche [40].

Typically mammals consume milk only during infancy, which is a period of very rapid growth and maturation, and each species' milk reflects the particular growth pattern and needs of its infants. Milk contains calories, vitamins and minerals, and specific biochemicals that enhance growth and development above and beyond its nutrients and energy [41]. However, milk – indeed that of another species with a different life history – is now widely consumed by many humans well beyond the traditional age at weaning. Milk comes most frequently from bovine species (especially cows, *Bos spp.*), which have a radically different juvenile growth pattern than humans, i.e. calves grow faster and to much larger sizes [42], [43]. The question thus arises as to whether consumption of a growth-promoting substance from another species throughout childhood fundamentally alters processes of human growth and maturation.

IGF-I is part of the protein fraction of milk, structurally similar to insulin, and a powerful mitogen that stimulates growth in a variety of tissues. IGF-I is molecularly identical in bovine and human milk and the concentration does not vary substantially between the species' milks [44], [45]. Animal protein in general and milk in particular have been found to increase circulating levels of IGF-I [38], [46], [47], [48]. It remains unclear as to whether the IGF-I in milk is itself responsible for the increase in IGF-I in circulation or if milk stimulates endogenous IGF-I production [46]. Debate continues over whether any IGF-I is absorbed intact or if it is fully digested in the gastrointestinal tract in post-weaning age children or adults [48], [49].

IGF-I is associated with rapid growth [50], child height [47], [51], and pubertal development [52], [53]. Milk consumption, IGF-I levels, and height have been shown to be positively correlated among children [47], [51]. Current milk intake is not strongly associated with growth in height among U.S. children age 6–11 yrs [54], although it is positively associated with height and BMI in preschool children [55], [56], and height among adolescents [54], [57]. Dairy products have not been found to have the same associations with growth in height [55], [56]. Many concerns have been raised about whether the use of synthetic bovine growth hormone (rBGH or rBST) in cows to boost milk yield increases IGF-I concentrations in milk and thereby accelerates growth and/or sexual maturation. Monsanto researchers have reported that there is no difference in IGF-I concentrations in commercially available conventional milk, milk labeled as from cows not treated with rBGH, or organic milk, which by definition cannot be from cows treated with rBGH [58]. The Food and Drug Administration's conclusion is that milk from rBGH-treated cows is safe for human consumption and that IGF-I levels are not elevated compared to either human milk or non-rBGH-treated cow milk and IGF-I is digested and hence not directly absorbed [49]. Currently only a small minority of cows in the U.S. are treated with rBGH and many major supermarket brands of milk are from farmers who pledge not to use rBGH (Mike Schutz, personal communication; a precise accounting is not publically available).

Given that milk consumption is strongly encouraged and widely promoted for children in the United States [59], [60], and milk nutrients and IGF-I are both associated with somatic growth and pubertal development, it is valuable to ascertain whether milk during childhood promotes earlier sexual maturation. To do so, this study tests the following hypotheses: greater reported milk intake frequency in childhood will be associated with a lower age at menarche, or increased probability of early menarche, defined as <12 years [20] among U.S. women age 20–49 yrs, and greater milk or total dairy intake and reported frequency of milk consumption will be associated with an increased likelihood of early menarche in U.S. girls age 9–12 yrs.

## Materials and Methods

Data come from the publically-available 1999-2004 National Health and Examination Survey (NHANES) [61], an annual survey of health, diet, and nutritional status of the civilian non-institutionalized U.S. population. Each survey covers ∼5000 individuals across the age spectrum, and has a complex, multistage sampling design. The 1999–2004 samples were merged for this analysis, representing six cycles of data collection. STATA 10.0's survey data analysis tool was used in all analyses [62]. As per the NHANES analytic guidelines [63], sample weights were constructed for the six year composite sample. For women, the sample weight was based on those participating in the interview; for girls, it was based on those who completed the 24-h diet recall and examination. All results presented here were based on the weighted samples.

All females in NHANES 1999–2004 age 12+ years were asked about their age at menarche during the interview. This was reported in whole years, and so cannot be used as a measure of their exact menarcheal age or to calculate a precise average age at menarche for U.S. women. Available covariates that could potentially have associations with age at menarche included: age, ethnicity, education (less than high school, high school completion, post-high school education, used here as a rough index of childhood socioeconomic status, the only estimate available for adults in NHANES), and whether or not the respondent had been born in the United States. For this analysis only individuals self-identifying as Non-Hispanic White, Non-Hispanic Black, and Mexican-American were included, due to the lack of specificity and small samples sizes of the other two categories (“Other Hispanic” and “Other”) and research showing differences in age at menarche among these three groups. Although members of these groups are diverse and the groupings are not biologically comparable, these are the labels used in NHANES. They will be referred to in this paper as “ethnic groups.”

In 1999–2000 all adults age 20+ were asked about the frequency of their milk intake at ages 5–12 years. This became a gated question in 2001; adults were first asked whether they had been regular milk consumers. Regular milk consumption was defined as at least 5 times per week. Only those who reported being regular or “variable” milk drinkers were then asked specific questions about the frequency of their milk intake at 5–12 yrs. For this analysis a composite variable was constructed as a measure of milk intake during childhood. The following scale was used: 0 =  not a regular milk drinker or answered “never” or “rarely” to the question “How often did you drink any type of milk (including milk added to cereal) when you were a child between the ages of 5 and 12 years old;” 1 = sometimes or “it varied;” 2 =  daily. Consumption of all dairy products during childhood was not assessed for adults in NHANES, so the analysis of adult women will focus on milk only.

Out of 4264 women age 20–49 years, 3840 were of the three ethnic groups, reported whether they were born in the U.S., and had information on education. Of these 3188 reported their age at menarche and within this group 2657 reported their childhood milk consumption. The sample of 2657 women included for analysis here did not differ in age at menarche, education, or frequency of childhood milk intake from the larger set of NHANES women in this age group. Fewer women in the sample were born outside of the United States (10% vs. 16%), and as noted, the sample was restricted to the three ethnic groups. No additional information is available about when a woman migrated to the U.S, or the country of origin (aside from Mexico). The sample was limited to women age 20–49 years to avoid cohort effects on menarche age and milk consumption and recall inaccuracies with advancing age. There was no association between current age among women in this sample and their reported age at menarche or frequency of childhood milk intake.

For girls in NHANES, 8–11 year-olds were asked only if they had reached menarche yet while those age 12+ were also asked for their age at menarche if they had reached it. No 8 year-olds with information on covariates of interest reported attaining menarche, so the sample was restricted to those age 9 yrs and older. A 24-h recall provided data on milk and dairy intake and participants were queried about the frequency of milk intake over the past 30 days. Since both the amount and frequency of milk consumption decline markedly during adolescence in the NHANES sample, older adolescents who reported their age at menarche cannot be used to assess milk-menarche relationships as current intake is not a reliable measure of pre-menarcheal intake. Therefore the sample was restricted to 9–12 yr olds as milk and dairy intake (milk g, dairy kcal, daily vs. non-daily milk intake in the past 30 days) did not vary by age within this age range.

There were 1308 girls who were at least 108 months and less than 156 months of age (9–12 yrs, inclusive). Of these, 1209 belonged to one of the 3 ethnic groups, 1076 had birth weight and BMI information, 1048 reported their menarche status, and of those, 1008 completed at least day 1 of the 24-h recall and reported their frequency of milk intake over the past 30 days. Girls in the sample did not differ from the whole NHANES population of girls in this age group with respect to their likelihood of early menarche, BMI, birth weight, milk intake from the 24-h recall, or the frequency with which they reported daily milk consumption.

NHANES provides a full nutrient analysis of all foods consumed and total nutrient intake from the 24-h recall. For this analysis all types of fluid milk (plain and flavored milk, buttermilk, and reconstituted powdered milk) were aggregated to provide total beverage milk intake. A summary measure of other dairy product intake (yogurt, cheese, and ice cream) was also calculated from the 24-h recall. The 30-day milk consumption frequency variable was recoded into a dichotomous variable (daily vs. less frequent intake), due to the low number of girls who reported never or rarely drinking milk (n = 55, 4.7% of sample).

All results are reported as means (SE) or proportions of the sample. *Χ* ^2^ tests were used to determine differences in frequencies across categories, and linear regression with categorical variables was used to assess mean differences among milk or dairy categories. For adult women, linear regression was used to ascertain the contribution of milk intake frequency at 5–12 years to variation in reported age at menarche, controlling for other covariates including ethnicity, education, and whether or not the respondent was born in the U.S. Cox regression was employed to assess the risk of reaching menarche before age 12 using the same covariates.

For girls age 9–12 yrs Cox regression was also used to ascertain whether frequency or amount of milk intake or total dairy kcal affected the probability of reaching menarche before age 12 yrs. Cox regression allows for censored data, and so girls under age 12 yrs who had not yet reached menarche can be included in the analysis. This method of analysis has been used with other cross-sectional datasets, including NHANES III, to compare risk of menarche among girls age 10–16 [28], and in analyses of entry into menopause [64], [65]. Here age is a measure of “time at risk” and the data are right-censored (e.g. the 9 yr olds have not made it through the entire age period of risk). Ethnicity, birth weight, total kcal intake (from the 24-h recall) were used as covariates in the base model Cox regression. Overweight status (BMII≥85^th^ percentile; less than 3% of the sample was underweight [<5^th^%ile for BMI] and none of those girls reported early menarche, so the underweight category was combined with the normal weight group) and height percentiles (<25^th^ percentile; 25^th^–75^th^ percentile, ≥75^th^ percentile), percentiles based on the WHO/NCHS standard [66] were added to see if they mediated any relationship between milk and risk of early menarche. Hazard ratios for tertiles of milk intake (g), total dairy intake (kcal), dairy protein (g), dairy calcium (mg), dairy fat (g), total fat (g) and total calcium (mg), along with daily/less frequent 30-day milk intake and milk consumption in the past 24 h (none vs. any amount), overweight status, and height percentile grouping were calculated.

## Results

### Women age 20-49 yrs

Sample characteristics are presented in **Table 1**. Most women reported daily milk intake from ages 5–12 yrs; only 8% reported drinking it rarely or never. This varied by ethnicity and immigration status. Many fewer White women reported drinking milk rarely or never as children than Mexican-Americans or Blacks, while women born outside the U.S. were more likely to report rarely/never drinking milk than those born in the U.S. Black women reported earlier menarche than Whites, and had the highest frequency of early menarche. Women born in the U.S. had earlier menarche than those born elsewhere. Education had no relationship to milk consumption or age at menarche and was dropped as a covariate in subsequent analyses.

**Table 1:** Sample characteristics of women age 20–49 years.

	Total	Average	% drinking milk	Mean age at	% menarche
	sample	age (SE)	rarely/never	menarche (SE)	<12 yrs
Total sample (n = 2657)		35.2 (0.2)	8.4%	12.6 (0.03)	20.8%
milk consumption age 5–12 yrs					
rarely/never	8.4%	34.8 0.6)		12.9 (0.16)	17.9%
sometimes/varied	9.1%	33.8 (0.7)		12.6 (0.11)	20.7%
daily	82.5%	35.4 (0.2)		12.6 (0.04)	21.1%
Ethnic group					
White	76.8%	35.5 (0.3)	6.8%‡	12.7 (0.04)	18.7%‡
Mexican-American	9.6%	32.8 (0.3)	16.1%	12.5 (0.08)	24.6%
African-American	13.6%	34.9 (0.3)	12.1%	12.4 (0.08)‡	30.0%
Born in USA?					
yes	89.7%	34.1 (0.5)	16.1%‡	12.6 (0.04)	21.2%
no	10.3%	35.3 (0.2)	7.5%	12.9 (0.08)†	17.4%

† p<0.01;

‡ p<0.001.

Regression coefficients for predicting age at menarche (in years) are presented in **Table 2**. Mexican-Americans and Blacks had lower ages at menarche than Whites, and women who were born outside of the U.S. had higher ages at menarche than those born in the U.S. Differences among the groups were independent of milk intake during childhood. The association between frequency of milk consumption during childhood and age at menarche suggests a negative association, although differences between the “rarely/never” group (the referent group) and the “sometimes/variable” or daily intake groups were statistically significant only at *P*<0.061 and 0.095, respectively.

**Table 2:** Predictions for age at menarche from multivariate regression and odds ratios for menarche<12 yrs from logistic regression among women 20–49 yrs.

	β (change in	95% CI	*P*	Hazard Ratio	95% CI	*P*
	menarche age, yrs)			Menarche <12 yrs		
Ethnic GroupWhite (reference group)						
Mexican-American	−0.398	(−0.65, −0.15)	0.002	1.78	(1.31, 2.41)	0.001
Black	−0.338	(−0.52, −0.16)	0.000	1.78	(1.47, 2.16)	0.001
Born in the USA? (1 = yes)	−0.428	(−0.68, −0.18)	0.001	1.64	(1.12, 2.41)	0.013
Milk consumption 5–12 yrs						
rarely/never (reference group)						
sometimes/varied	−0.378	(−0.77, 0.02)	0.061	1.20	(0.76, 1.91)	0.422
daily	−0.317	(−0.69, 0.06)	0.095	1.25	(0.86, 1.81)	0.231

As shown in Table 2, in Cox regression there was no difference in the risk of menarche before 12 yrs between those reporting drinking milk “sometimes/varied” (HR: 1.20, *P*<0.42) or daily (HR: 1.25, *P*<0.23) and those who rarely or never consumed milk. The same pattern of ethnic group differences was found for early menarche, with Blacks and Mexican-Americans almost twice as likely as Whites to report early menarche. Those born in the U.S. had greater odds of reaching early menarche than those born elsewhere.

### Girls age 9-12 yrs

Characteristics of the sample of girls age 9–12 years are in **Table 3**. Almost 19% of all girls in this age group reported that they had reached menarche prior to age 12, although since many were still below age 12, this is not the true rate of early menarche in the sample. Milk grams, total dairy kcal, total kcal, total calcium, and % overweight did not vary by age in the sample, with the exception of the 11 yr olds, who consumed less milk and dairy calcium than the 10 yr olds and less total calcium than all other ages. Girls who reported less than daily milk consumption consumed significantly less milk g, dairy calcium, and total calcium than those reporting daily consumption (all *P*<0.001). There were no significant differences in age among 3 ethnic groups, while milk and total dairy consumption did vary by ethnic group with Blacks consuming the least milk g and Whites consuming the most total dairy kcal. There were no ethnic differences in frequency of milk intake. Blacks were more likely to be overweight than Whites but not Mexican-Americans. Overweight girls reported drinking significantly less milk than those with lower BMIs, although 30 day frequency of milk consumption and total dairy kcal and other nutrients did not vary by overweight status. Girls with height <25^th^ percentile drank less milk than those with heights ≥75^th^ percentile.

**Table 3:** Characteristics of sample of girls 9–12 yrs, by age, ethnicity, dietary intake, menarche status and BMI percentile.

	total sample	drank milk	milk g	total dairy	dairy	dairy calcium	total	calcium	percent
	(SE)	daily	(SE)	kcal (SE)	fat g (SE)	mg (SE)	kcal (SE)	mg (SE)	overweight
Total sample (n = 1008)		79.7%	191 (14)	214 (14)	10.4 (0.6)	350 (23)	1959 (35)	913 (33)	43%
Age									
−9 yrs	24.4%	88.5%	201 (21)	221 (21)	11.0 1.3)	369 (33)	1986 (58)	936 (56)	43%
−10 yrs	22.6%	80.4%	219 (30)	225 (22)	10.8 (1.2)	392 (40)	2065 (97)	1036 (90)	49%
−11 yrs	28.5%	75.0%	149 (24)^b^	192 (24)	8.7 (1.1)	282 (37)^b^	1838 (80)	777 (42)^c^	42%
−12 yrs	24.6%	75.9%^a^	203 (26)	224 (23)	10.6 (1.1)	374 (38)	1977 (530	934 (43)	40%
Ethnic group									
White	69.2%	79.9%	206 (19)	231 (19)*	10.8 (0.9)	377 (31)	1946 (47)	941 (47)	40%
Mexican-American	14.1%	85.4%	183 (14)	186 (12)	9.1 (0.7)	324 (24)	2000 (53)	934 (46)	44%
Black	16.7%	74.2%	138 (12)*	171 (13)	8.4 (0.7)*	263 (18)†	1983 (50)	778 (27)†	54%*
Milk consumption in past 30 days									
never/rarely/sometimes/varied	20.3%		89(14)	142(20)	7.4 (1.1)	201 (26)	1821 (87)	681 (38)	39%
Daily	79.7%		217 (15)‡	233 (14)‡	10.9 (0.7)†	388 (24)‡	1995 (38)	972 (36)‡	44%
height <25^th^ %ile	16.8%	81.1%	245 (41)*	259 (40)	12.6 (2.2)	435 (72	1896 (84)	922 (73)	16%
height ≥25^th^ %ile & <75^th^ %ile	43.6%	78.7%	194 (20)	197 (14)	9.3 (0.8)	328 (27)	1880 (88)†	852 (40)	40%
height ≥75^th^ %ile	39.6%	80.3%	165 (14)	214 (15)	10.1 (0.8)	340 (26)	2073 (56)	975 (55)	58%
overweight (BMI≥85^th^ %ile)	43.0%	81.6%	168 (15)	212 (18)	10.3 (0.8)	338 (25)	1899 (41)	865 (38)	
normal/under weight (BMI <85th %ile)	57.0%	78.30%	209 (17)*	216 (16)	10.1 (0.8)	360 (28)	2004 (55)	949 (42)	

*P<0.05;

† *P*<0.01;

‡ *P*<0.001.

a X^2^ for age groups *P*<0.09;

b 11 yr olds different from 10 yr olds, *P*<0.04;

c 11 yr olds lower than all other age groups *P*<0.03.

As shown in **Table 4**, after controlling for birth weight, ethnic group and total kcal, total dairy kcal, protein or calcium, and frequency of milk intake over the past 30 days each had no association with the risk of menarche prior to 12 yrs in Cox regression. The only associations were with milk g intake from the 24-h recall, dairy fat, and total calcium. Girls in the middle tertile of milk g intake had a lower risk of early menarche than those in the highest tertile after controlling for birth weight, ethnicity, and total energy intake, although significance was marginal (HR: 0.6, *P*<0.06) and there was no difference in risk between the highest and lowest tertiles. Those in the lowest tertiles of dairy fat intake had an increased risk of early menarche compared those in the highest tertile (lowest tertile HR: 1.5, *P*<0.05; middle tertile HR: 1.6, *P*<0.07 compared to the highest). A lower risk of early menarche was found among girls in the middle tertile of calcium intake (HR: 0.6, *P*<0.05) than those in the highest tertile. Controlling for milk intake, total calcium, and dairy fat in the same equation resulted in the same pattern (milk intake second tertile HR = 0.6, *P*<0.04; calcium in tertile I HR = 0.6, *P*<0.006; dairy fat HR = 2.2, *P*<0.005 for lowest tertile and HR = 1.8, *P*<0.01 for middle tertile. All compared to highest tertiles). Total fat, protein, kcal, and birth weight were not related to risk of early menarche. Blacks had a higher risk of early menarche than Whites (HR: 1.6, *P*<0.03) but not Mexican-Americans.

**Table 4:** Hazard ratios (HRs) for reaching menarche <12 years, girls 9–12 yrs.

	HR (95% CI)	HR (95% CI)	HR (95% CI)
		Tertile I	Tertile II
Milk g tertiles (0, 0–244, 245+)		0.8 (0.5, 1.3)^NS^	0.6 (0.4, 1.0)^P<0.06^
Dairy kcal tertiles (0–99, 100–229, 230+ kcal)		1.2 (0.7, 2.0)^NS^	1.2 (0.7, 1.9)^NS^
Dairy calcium tertiles (0–199, 200–399, 400+ mg)		1.0 (0.6, 1.7)^NS^	0.9 (0.6, 1.3)^NS^
Dairy protein tertiles (<6, 6–11.99, 12+ g)		0.9 (0.6, 1.5)^NS^	0.7 (0.5, 1.1)^NS^
Dairy fat tertiles (<4, 4–9.99, 10+ g)		1.5 (1.0, 2.5)^p<0.07^	1.6 (1.0, 2.4)*
Total calcium tertiles (0–599, 600–999, 1000+)		0.6 (0.3, 1.0)*	0.8 (0.5, 1.2)^NS^
Daily vs. never/rarely/sometimes/varied			
milk consumption in the past 30 days	1.0 (0.6, 1.6)^NS^		

a Highest tertile and White are reference categories;

*P<0.05.

**Base model hazard ratios (**95% CI): birth weight: 1.0 (0.7, 1.4, NS), total kcal: 1.00 (1.0, 1.0, NS); Mexican-American 1.5 (0.8, 2.7, NS); Black 1.6 (1.0, 2.6, *P*<0.03).

These results remained after controlling for overweight status (**Table 5**). Overweight girls had a higher risk of early menarche (HR: 2.2, *P*<0.001) than those with lower BMIs, and controlling for overweight eliminated the difference in hazard ratios between Blacks and Whites. **Table 6** adjusts further for height percentile. Girls with heights ≥75^th^ percentile were more likely to have early menarche than those in the mid-range of height (25^th^–75^th^ percentile) or those with heights <25^th^ percentile (HR: 2.5, *P*<0.001 and HR: 5.6, *P*<0.001, respectively). Those in the lowest height percentile group did not have a lower risk of early menarche than those in the mid-range although the *p*-value was close to significance (HR: 0.4, *P*<0.06). Overweight status remained a significant predictor of early menarche after controlling for height (HR: 1.8, *P*<0.01) and results for the dairy variables and milk intake remained virtually unchanged.

**Table 5:** Hazard ratios (HRs) for reaching menarche <12 years among girls 9–12 yrs, adjusting for overweight status.

	HR (95% CI)	HR (95% CI)	HR (95% CI)
		Tertile I	Tertile II
Milk g tertiles (0, 0–244, 245+)		0.8 (0.5, 1.3)^NS^	0.6 (0.4, 1.0)*
Dairy kcal tertiles (0–99, 100–229, 230+ kcal)		1.4 (0.8, 2.3)^NS^	1.3 (0.8, 2.1)^NS^
Dairy calcium tertiles (0–199, 200–399, 400+ mg)		1.1 (0.6, 1.8)^NS^	1.0 (0.6, 1.5)^NS^
Dairy protein tertiles (<6, 6–11.99, 12+ g)		1.0(0.6, 1.7)^NS^	0.8 (0.5, 1.2)^NS^
Dairy fat tertiles (<4, 4–9.99, 10+ g)		1.8 (1.2, 2.9)†	1.9 (1.2, 3.0)†
Total calcium tertiles (0–599, 600–999, 1000+)		0.6 (0.3,1.0)^P<0.07^	0.7 (0.5, 1.3)^NS^
Daily vs. never/rarely/sometimes/varied			
milk consumption in the past 30 days	0.9 (0.6, 1.6)^NS^		

a Highest tertile and White are reference categories.

*P<0.05;

† *P*<0.01.

**Base model hazard ratios** (95% CI): birth weight: 0.9 (0.7,1.2, NS), total kcal: 1.00 (1.0, 1.0, p<0.06); Mexican-American 1.3 (0.7, 2.5, NS); Black 1.4 (0.9, 2.2, NS); overweight 2.2 (1.4, 3.4, *P*<0.001).

**Table 6:** Hazard ratios (HRs) for reaching menarche <12 years among girls 9–12 yrs, adjusting for overweight status and height percentile.

	HR (95% CI)	HR (95% CI)	HR (95% CI)
		Tertile I	Tertile II
Milk g tertiles (0, 0–244, 245+)		0.7 (0.5, 1.2)^NS^	0.6 (0.4, 1.0)*
Dairy kcal tertiles (0–99, 100–229, 230+ kcal)		1.4 (0.9, 2.2)^NS^	0.6 (0.4, 1.0)*
Dairy calcium tertiles (0–199, 200–399, 400+ mg)		1.0 (0.6, 1.7)^NS^	0.9 (0.6, 1.3)^NS^
Dairy protein tertiles (<6, 6–11.99, 12+ g)		1.0 (0.6, 1.6)^NS^	0.8 (0.5, 1.1)^NS^
Dairy fat tertiles (<4, 4–9.99, 10+ g)		1.9 (1.2, 2.9)†	2.0 (1.3, 3.1)†
Total calcium tertiles (0–599, 600–999, 1000+)		0.6 (0.3, 1.0)*	0.7 (0.4, 1.1)^P<0.09^
Daily vs. never/rarely/sometimes/varied			
milk consumption in the past 30 days	0.9 (0.6, 1.4)^NS^		

a Highest tertile and White are reference categories.

*P<0.05;

† *P*<0.01.

**Base model hazard ratios** (95% CI): birth weight: 0.8 (0.6,1.1, NS), total kcal: 1.00 (1.0, 1.0, NS); Mexican-American 1.5 (0.8, 2.9, NS); Black 1.2 (0.8, 1.8, NS); overweight 1.8 (1.2, 2.7; *P*<0.01); height <25^th^ %ile 0.4 (0.2, 1.0, *NS*), height≥75^th^ %ile 2.5 (1.5, 4.0, *P*<0.001).

With milk g, dairy fat, and total calcium in the model, overweight girls retained a greater risk (HR:2.2, 95% CI: 1.4, 3.3, *P*<0.001) for menarche <12 yrs compared to girls with lower BMIs, and girls ≥75^th^ percentile for height continued to have a higher risk for early menarche than those below the 25^th^ percentile (HR: 5.4, 95% CI: 2.5, 11.4, *P*<0.001) and those in the 25^th^–75^th^ percentile (HR: 2.5, 95% CI: 1.6, 3.6, *P*<0.001). Milk g, dairy fat, and total calcium each remained significant; girls in the lowest two tertiles of dairy fat intake had higher risks of early menarche than those with greater dairy fat intake (HR: 2.7 95% CI: 1.8, 3.9, *P*<0.001, HR: 2.2 95% CI: 1.4, 3.5, for lowest and middle tertiles, respectively; *P*<0.001 for both). Similarly, girls in the lowest tertiles of total calcium intake had lower risk of early menarche compared to those with the greatest calcium intake (HR: 0.4 95% CI: 0.3, 0.8, *P*<0.004; HR: 0.6, 95% CI: 0.4, 1.0, *P*<0.05, for lowest and middle tertiles, respectively). The middle tertile of milk intake continued to be associated with a lower risk of early menarche than the highest tertile (HR: 0.6, 95% CI: 0.4, 0.9, *P*<0.02).

## Discussion

A primary finding of this study was a weak negative association between frequency of milk intake during childhood and age at menarche for U.S. women age 20–49. Controlling for the same covariates (ethnic group, whether the woman was born in the U.S.), milk consumption at 5–12 yrs was similarly weakly related to height in this sample (β = 0.84, *P*<0.06) and height and age at menarche were positively associated (*P*<0.001). These consistent results suggest that inaccuracy in recall of age at menarche is not likely to be responsible for the relationship between milk intake and age at menarche. There was no difference in the risk of early menarche among women reporting different frequencies of milk intake during childhood. Likewise, among 9–12 year old girls there was no relationship between frequency of milk intake in the past 30 days and early menarche.

There was a negative relationship between quantity of milk consumed in the past 24 hrs and risk of early menarche for girls 9–12 yrs. Girls in the second tertile of milk intake had a lower risk of early menarche than did those in the highest tertile. This association was strengthened after controlling for height as well as total calcium and dairy fat; thus milk's relationship to early menarche is not mediated by associations between milk consumption and childhood height or calcium or dairy fat. Total calcium had a positive effect on the risk of early menarche, while greater dairy fat intake was associated with a lower risk of early menarche, associations that remained after controlling for overweight, height percentile, and total milk intake. Other studies had suggested that greater intake of milk or milk-related nutrients such as calcium, protein, or fat contributed to earlier menarche [30], [31], [32]}. In this study only greater total calcium and milk intake contributed to a higher risk of early menarche in this sample from NHANES 1999–2004. Calcium intake was correlated with overall dairy intake (r = 0.20, *P*<0.001) and milk intake (r = 0.60, *P*<0.001), although on average only 35% of total calcium intake came from dairy products.

The finding that greater dairy fat intake was associated with a lower risk of early menarche was unexpected but it was a consistent and strong finding. It may be that dairy fat is strongly correlated with an unmeasured variable that is related to early menarche. It was significantly higher among girls reporting daily milk intake, and this may be a marker for other behaviors that are themselves associated with later menarche.

Ethnic differences in age at menarche were consistent with other surveys [27], [30] and were independent of differences in milk intake. Among adult women, Blacks and Mexican-Americans were more likely to reach menarche early, but in the sample of girls 9–12 yrs only Blacks had a higher risk of early menarche than Whites, and this disappeared after controlling for overweight, suggesting that the variation in early menarche was mediated by ethnic differences in BMI.

Similar to other studies [14], [18], [19], [20], [21], [67], overweight and greater height were associated with higher probabilities of early menarche. Overweight girls had roughly twice the risk of early menarche than those of lower BMIs; girls at or above the 75^th^ percentile for height had a risk that was 1.5 times those between the 25^th^ and 75^th^ percentiles and 5.5 times those with heights less than the 25^th^ percentile. Height and BMI percentiles were strongly positively correlated in this study (r = 0.30, *P*<0.001), but the two independently predicted risk of early menarche. Although these measures were assessed at the girl's current age, which for 18.6% of the sample was post-menarche, the use of broad categories (overweight vs. normal/underweight and 3 height groups) rather than individual specific percentiles reduced the likelihood that their index changed after achieving menarche. Studies have found that BMI and height are not greater for up to 4 yrs post-menarche for girls with early menarche compared with those with later menarche [68], [69] and that BMI is affected by other pubertal markers but not menarcheal status among adolescent girls [70]. Others have shown that greater height and BMI are evident in early menarcheal girls pre- but not post-menarche [22], that body fat does not change with menarcheal status among peri-menarcheal girls [71], and that girls are likely to stay in their same percentile of fatness as they mature [72].

Also consistent with some studies of older children [73], [74], [75], but not others [38], [76], [77], was a negative association between milk intake and overweight although here the relationship was limited to milk intake and not to overall dairy intake, calcium, or milk consumption frequency. Controlling for overweight and height did not change the relationship between milk and early menarche, suggesting that underlying connections between milk and body size are not mediating the relationship between milk and early menarche.

Previous analyses of NHANES data suggest that milk consumption in earlier childhood is associated with both greater height and BMI, after controlling for birth weight [55], [56], although milk intake in the ages leading up to menarche (6–11 yrs) is not associated with height [54]. Rapid early growth or gains in BMI, especially when preceded by impaired fetal growth, are associated with earlier menarche [15], [22], [24], [32], [78], and so milk consumption in early childhood may contribute to accelerated menarche by upregulating early growth in body size. Thus milk may be related to menarche through its contributions to somatic growth and other mechanisms related to reproductive maturation. Or it may act via a common pathway (e.g. IGF-I, although dairy protein was not related to early menarche in this study), as IGF-I is involved in both somatic growth and reproductive maturation [79]. IGF-I has been correlated with milk and calcium intake, but not with fat intake among adolescent girls [80]. Longitudinal studies that follow young children's growth, milk consumption, and subsequent sexual maturation and IGF-I are necessary to assess these relationships.

Dairy intake may be a marker for other dietary behaviors or related to physical activity, which was not evaluated here. NHANES does provide data on physical activity, but it is measured differently for children <12 yrs and 12+ yrs, and thus it could not be used for this sample. If dairy intake is correlated with physical activity, particularly strenuous exercise, this may contribute to changes in BMI and menarche. However, surveys of female adolescent athletes suggest no difference in dairy intake with non-athletes [81] or lower intake due to concerns about weight gain [82], [83].

Other limitations of this study include the fact that NHANES is a cross-sectional study, so causal relationships cannot be determined; the need to use current dietary data for girls, which may not be an accurate marker of long term intake in childhood; the use of current height and BMI, which for almost 19% of girls was post-menarche; reliance on a single 24-h recall, which is subject to both under- and over-reporting and which may not be indicative of overall dietary habits [84], [85]; and the potential correlations between milk intake and other characteristics that may influence age at menarche. In addition, adult data on milk consumption and age at menarche are based on recall of events that may be quite distant in the past (e.g. the oldest women in the study are recalling their milk intake from age 5–12 yrs, which is 37–44 yrs prior). Other researchers have used these data to gauge the relationship between childhood milk intake and fracture rates in post-menopausal women [86]. On the whole, studies that assess the match between recall of dietary behavior in the distant past and reported intake from historical questionnaires have reported a range of results from high to low correlations, including for milk or dairy products specifically [87]. Furthermore, some studies report that recall diminishes with age, while others report no age effect [87]. Recall of menarcheal age may also be faulty for women, although one study showed that it was equally likely to be overestimated by 1 year as underestimated by 1 year [88].

In sum, cows' milk may be a unique food in the human diet in its capacity to alter life history trajectories as they unfold during childhood. Its ability to do so appears unrelated or in addition to its macronutrient profile (e.g. calcium). Dairy products, in which bioactive milk ingredients are altered or rendered inactive, may not have the same effects. It may be that milk consumption, which evolutionarily would have been limited to very young children, sends physiological signals that regulate somatic growth and development as well as reproductive maturation, and insofar as somatic growth in height or BMI is related to age at menarche, milk consumption may have both immediate as well as indirect or longer-term impacts on the timing of sexual maturation.

## References

[1] Parent A-S, Teilmann G, Juul A, Skakkebaek NE, Toppari J (2003). The Timing of Normal Puberty and the Age Limits of Sexual
Precocity: Variations around the World, Secular Trends, and Changes after
Migration.. Endocr Rev.

[2] Wehkalampi K, Silventoinen K, Kaprio J, Dick DM, Rose RJ (2008). Genetic and environmental influences on pubertal timing assessed
by height growth.. Am J Hum Biol.

[3] Kaprio J, Rimpela A, Winter T, Viken RJ, Rimpela M (1995). Common genetic influences on BMI and age at
menarche.. Hum Biol.

[4] Frisch RE, Revelle R (1970). Height and weight at menarche and a hypothesis of critical body
weights and adolescent events.. Science 169.

[5] Ellison PT (1994). Advances in Human Reproductive Ecology.. Annu Rev Anthrop.

[6] Ellison PT (2003). Energetics and reproductive effort.. Amer J Hum Biol.

[7] Allsworth JE, Weitzen S, Boardman LA (2005). Early age at menarche and allostatic load: data from the third
National Health and Nutrition Examination Survey.. Ann Epidemiol.

[8] Ellis BJ (2004). Timing of pubertal maturation in girls: an integrated life
history approach.. Psychol Bull.

[9] Chisholm JS (1993). Death, Hope, and Sex: Life-history theory and the development of
reproductive strategies.. Curr Anthrop.

[10] Belsky J, Steinberg L, Draper P (1991). Childhood experience, interpersonal development, and reproductive
strategies: An evolutionary theory of socialization.. Child Dev.

[11] Eveleth PB, Tanner JM (1990). Worldwide variation in human growth..

[12] Wyshak G, Frisch RE (1982). Evidence for a secular trend in age of menarche.. N Engl J Med.

[13] Ong KK, Ahmed ML, Dunger DB (2006). Lessons from large population studies on timing and tempo of
puberty (secular trends and relation to body size): The European
trend.. Mol Cell Endocrinol.

[14] Kaplowitz PB (2008). Link between body fat and the timing of puberty.. Pediatrics.

[15] Dunger DB, Ahmed ML, Ong KK (2006). Early and late weight gain and the timing of
puberty.. Mol Cell Endocrinol.

[16] Kulin HE, Bwibo N, Mutie D, Santner SJ (1982). The effect of chronic childhood malnutrition on pubertal growth
and development.. Am J Clin Nutr.

[17] Kaplowitz P (2006). Pubertal development in girls: secular trends.. Curr Opin Obstet Gynecol.

[18] Rosenfield RL, Lipton RB, Drum ML (2009). Thelarche, pubarche, and menarche attainment in children with
normal and elevated body mass index.. Pediatrics.

[19] Wattigney WA, Srinivasan SR, Chen W, Greenlund KJ, Berenson GS (1999). Secular trend of earlier onset of menarche with increasing
obesity in black and white girls: the Bogalusa Heart Study.. Ethn Dis.

[20] Adair LS (2001). Size at Birth Predicts Age at Menarche.. Pediatrics.

[21] Bratberg G, Nilsen T, Holmen T, Vatten L (2007). Early sexual maturation, central adiposity and subsequent
overweight in late adolescence. A four-year follow-up of 1605 adolescent
Norwegian boys and girls: the Young HUNT study.. BMC Public Health.

[22] Salsberry PJ, Reagan PB, Pajer K (2009). Growth differences by age of menarche in African American and
White girls.. Nurs Res.

[23] Khan AD, Schroeder DG, Martorell R, Rivera JA (1995). Age at menarche and nutritional supplementation.. Journal of Nutrition.

[24] Silva IdS, Stavola BL, Mann V, Kuh D, Hardy R (2002). Prenatal factors, childhood growth trajectories and age at
menarche.. Int J Epidemiol.

[25] Tam CS, de Zegher F, Garnett SP, Baur LA, Cowell CT (2006). Opposing influences of prenatal and postnatal growth on the
timing of menarche.. J Clin Endocrinol Metab.

[26] Biro FM, Huang B, Crawford PB, Lucky AW, Striegel-Moore R (2006). Pubertal correlates in black and white girls.. J Pediatr.

[27] Chumlea WC, Schubert CM, Roche AF, Kulin HE, Lee PA (2003). Age at menarche and racial comparisons in US
girls.. Pediatrics.

[28] Wu T, Mendola P, Buck GM (2002). Ethnic differences in the presence of secondary sex
characteristics and menarche among US girls: the Third National Health and
Nutrition Examination Survey, 1988-1994.. Pediatrics.

[29] Geronimus AT, Bound J, Waidmann TA (1999). Health inequality and population variation in fertility
timing.. Soc Sci Med.

[30] Koo MM, Rohan TE, Jain M, McLaughlin JR, Corey PN (2002). A cohort study of dietary fibre intake and
menarche.. Public Health Nutrition.

[31] Chevalley T, Rizzoli R, Hans D, Ferrari S, Bonjour J-P (2005). Interaction between calcium intake and menarcheal age on bone
mass gain: An eight-year follow-up study from prepuberty to
postmenarche.. J Clin Endocrinol Metab.

[32] Berkey CS, Gardner JD, Frazier AL, Colditz GA (2000). Relation of childhood diet and body size to menarche and
adolescent growth in girls.. Am J Epidemiol.

[33] Gunther ALB, Karaolis-Danckert N, Kroke A, Remer T, Buyken AE (2010). Dietary Protein Intake throughout Childhood Is Associated with
the Timing of Puberty.. J Nutr.

[34] Sanchez A, Kissinger DG, Phillips RI (1981). A hypothesis on the etiological role of diet on age at
menarche.. Med Hypotheses.

[35] Colditz GA, Willett WC, Stampfer MJ, Rosner B, Speizer FE (1987). A prospective study of age at menarche, parity, age at first
birth, and coronary heart disease in women.. Am J Epidemiol.

[36] Moisan J, Meyer F, Gingras S (1990). Diet and age at menarche.. Cancer Causes Control.

[37] Petridou E, Syrigou E, Toupadaki N, Zavitsanos X, Willett WC (1996). Determinants of age at menarche as early life predictors of
breast cancer risk.. Int J Cancer.

[38] Cadogan J, Eastell R, Jones N, Barker ME (1997). Milk intake and bone mineral acquisition in adolescent girls:
Randomised, controlled intervention trial.. Br Med J.

[39] Du X, Zhu K, Trube A, Zhang Q, Ma G (2004). School-milk intervention trial enhances growth and bone mineral
accretion in Chinese girls aged 10-12 years in Beijing.. Br J Nutr.

[40] Zhu K, Zhang Q, Foo LH, Trube A, Ma G (2006). Growth, bone mass, and vitamin D status of Chinese adolescent
girls 3 y after withdrawal of milk supplementation.. Am J Clin Nutr.

[41] Patton S (2004). Milk: Its Remarkable Contribution to Human Health and
Well-Being..

[42] Marlowe TJ, Gaines JA (1958). The Influence of Age, Sex, and Season of Birth of Calf, and Age
of Dam on Preweaning Growth Rate and Type Score of Beef
Calves.. J Anim Sci.

[43] Reynolds WL, DeRouen TM, Bellows RA (1978). Relationships of Milk Yield of Dam to Early Growth Rate of
Straightbred and Crossbred Calves.. J Anim Sci.

[44] Campana WM, Baumrucker CR, Jensen RG (1995). Hormones and growth factors in bovine milk.. Handbook of Milk Composition.

[45] Koldovsky O, Strbak V, Jensen RG (1995). Hormones and growth factors in human milk.. Handbook of Milk Composition.

[46] Holmes MD, Pollak MN, Willett WC, Hankinson SE (2002). Dietary correlates of plasma insulin-like growth factor I and
insulin-like growth factor binding protein 3 concentrations.. Cancer Epidemiology, Biomarkers, & Prevention.

[47] Hoppe C, Mølgaard C, Juul A, Michaelsen KF (2004). High intakes of skimmed milk, but not meat, increase serum IGF-I
and IGFBP-3 in eight-year-old boys.. Eur J Clin Nutr.

[48] Rich-Edwards JW, Ganmaa D, Pollak MN, Nakamoto EK, Kleinman K (2007). Milk consumption and the prepubertal somatotropic
axis.. Nutrition Journal.

[49] Juskevich JC, Guyer CG (1990). Bovine growth hormone: food safety evaluation.. Science.

[50] Fall CHD, Clark PM, Huindmarsh PC, Clayton PE, Shiell AW (2000). Urinary GH and IGF-I excretion in nine year-old children:
relation to sex, current size and size at birth.. Clin Endocrinol (Oxf).

[51] Rogers I, Emmett P, Gunnell D, Dunger D, Holly J (2006). Milk as a food for growth? The insulin-like growth factors
link.. Public Health Nutrition.

[52] Juul A, Bang P, Hertel NT, Main K, Dalgaard P (1994). Serum insulin-like growth factor-I in 1030 healthy children,
adolescents, and adults: Relation to age, sex, stage of puberty, testicular
size, and body mass index.. J Clin Endocrinol Metab.

[53] Juul A, Dalgaard P, Blum WF, Bang P, Hall K (1995). Serum levels of insulin-like growth factor (IGF)-binding
protein-3 (IGFBP-3) in healthy infants, children, and adolescents: the
relation to IGF-I, IGF-II, IGFBP-1, IGFBP-2, age, sex, body mass index, and
pubertal maturation.. J Clin Endocrinol Metab.

[54] Wiley AS (2005). Does milk make children grow? Relationships between milk
consumption and height in NHANES 1999-2002.. Amer J Hum Biol.

[55] Wiley AS (2009). Consumption of milk, but not other dairy products, is associated
with height among U.S. preschool children in NHANES
1999-2002.. Ann Hum Biol.

[56] Wiley AS (2010). Milk, but not dairy intake is positively associated with BMI
among U.S. children in NHANES 1999-2004.. Amer J Hum Biol.

[57] Berkey CS, Colditz GA, Rockett HRH, Frazier AL, Willett WC (2009). Dairy Consumption and Female Height Growth: Prospective Cohort
Study.. Cancer Epidemiology, Biomarkers, & Prevention.

[58] Vicini J, Etherton T, Kris-Etherton P, Ballam J, Denham S (2008). Survey of Retail Milk Composition as Affected by Label Claims
Regarding Farm-Management Practices.. J Am Diet Assoc.

[59] National Institutes of Health (1994). Optimal Calcium Intake.. Journal of the American Medical Association.

[60] United States Department of Health and Human Services, United States
Department of Agriculture (2005). Dietary Guidelines for Americans..

[61] Center for Disease Control/National Center for Health
Statistics (2007). National Health and Nutrition Examination Survey
Data..

[62] STATACorp Inc. (2008). STATA 10..

[63] Center for Disease Control/National Center for Health
Statistics (2006). The National Health and Nutrition Examination Survey (NHANES)
Analytic and Reporting Guidelines, Update: September, 2006..

[64] Luoto R, Kaprio J, Uutela A (1994). Age at Natural Menopause and Sociodemographic Status in
Finland.. Am J Epidemiol.

[65] Cramer DW, Harlow BL, Xu H, Fraer C, Barbieri R (1995). Cross-sectional and case-controlled analyses of the association
between smoking and early menopause.. Maturitas.

[66] de Onis M, Onyango AW, Borghi E, Siyam A, Nishida C (2007). Development of a WHO growth reference for school-aged children
and adolescents.. Bull World Health Organ.

[67] Maclure M, Travis LB, Willett WC, MacMahon B (1991). A prospective cohort study of nutrient intake and age at
menarche.. Am J Clin Nutr.

[68] Chang S-H, Tzeng S-J, Cheng J-Y, Chie W-C (2000). Height and Weight Change Across Menarche of Schoolgirls With
Early Menarche.. Arch Pediatr Adolesc Med.

[69] Demerath EW, Li J, Sun SS, Chumlea WC, Remsberg KE (2004). Fifty-year trends in serial body mass index during adolescence in
girls: the Fels Longitudinal Study.. Am J Clin Nutr.

[70] Bini V, Celi F, Berioli MG, Bacosi ML, Stella P (2000). Body mass index in children and adolescents according to age and
pubertal stage.. Eur J Clin Nutr.

[71] Legro RS, Lin HM, Demers LM, Lloyd T (2000). Rapid Maturation of the Reproductive Axis during Perimenarche
Independent of Body Composition.. J Clin Endocrinol Metab.

[72] Vink EE, van Coeverden SCCM, van Mil EG, Felius BA, van Leerdam FJM (2010). Changes and Tracking of Fat Mass in Pubertal
Girls.. Obesity.

[73] Barba G, Troiano E, Russo P, Venezia A, Siani A (2005). Inverse association between body mass and frequency of milk
consumption in children.. Br J Nutr.

[74] Novotny R, Daida YG, Acharya S, Grove JS, Vogt TM (2004). Dairy intake is associated with lower body fat and soda intake
with greater weight in adolescent girls.. Journal of Nutrition.

[75] Fiorito LM, Ventura AK, Mitchell DC, Smiciklas-Wright H, Birch LL (2006). Girls' dairy intake, energy intake, and weight
status.. J Am Diet Assoc.

[76] Berkey CS, Rockett HR, Willett WC, Colditz GA (2005). Milk, dairy fat, dietary calcium, and weight gain: a longitudinal
study of adolescents.. Arch Pediatr Adolesc Med.

[77] Phillips SM, Bandini LG, Cyr H, Colclough-Douglas S, Naumova E (2003). Dairy food consumption and body weight and fatness studied
longitudinally over the adolescent period.. Int J Obes Relat Metab Disord.

[78] Ingram CJ, Elamin MF, Mulcare CA, Weale ME, Tarekegn A (2007). A novel polymorphism associated with lactose tolerance in Africa:
multiple causes for lactase persistence?. Hum Genet.

[79] Juul A (2003). Serum levels of insulin-like growth factor I and its binding
proteins in health and disease.. Growth Horm IGF Res.

[80] Kerver JM, Gardiner JC, Dorgan JF, Rosen CJ, Velie EM (2010). Dietary predictors of the insulin-like growth factor system in
adolescent females: results from the Dietary Intervention Study in Children
(DISC).. Am J Clin Nutr.

[81] Perron M, Endres J (1985). Knowledge, attitudes, and dietary practices of female
athletes.. J Am Diet Assoc.

[82] Frederick L, Hawkins ST (1992). A comparison of nutrition knowledge and attitudes, dietary
practices, and bone densities of postmenopausal women, female college
athletes, and nonathletic college women.. J Am Diet Assoc.

[83] Beja-Pereira A, Luikart G, England PR, Bradley DG, Jann OC (2003). Gene-culture coevolution between cattle milk protein genes and
human lactase genes.. Nat Genet.

[84] Rockett HR, Berkey CS, Colditz GA (2003). Evaluation of dietary assessment instruments in
adolescents.. Curr Opin Clin Nutr Metab Care.

[85] Serdula MK, Alexander MP, Sacanlon KS, Bowman BA (2001). What are preschool children eating? A review of dietary
assessment.. Annu Rev Nutr.

[86] Kalkwarf HJ, Khoury JC, Lanphear BP (2003). Milk intake during childhood and adolescence, adult bone density,
and osteoporotic fractures in US women.. Am J Clin Nutr.

[87] Friedenreich CM, Slimani N, Riboli E (1992). Measurement of Past Diet: Review of Previous and Proposed
Methods.. Epidemiol Rev.

[88] Żarów R, Cichocka BA (2008). A comparative analysis of estimation of age at menarche by
various methods in women participating in the Krakow Longitudinal Growth
Study, Poland.. Amer J Hum Biol.

